# Psychosis-Proneness and Neural Correlates of Self-Inhibition in Theory of Mind

**DOI:** 10.1371/journal.pone.0067774

**Published:** 2013-07-18

**Authors:** Lisette van der Meer, Nynke A. Groenewold, Marieke Pijnenborg, André Aleman

**Affiliations:** 1 Department of Neuroscience, University Medical Center Groningen, Groningen, The Netherlands; 2 Department of Rehabilitation, Lentis Mental Health Care, Zuidlaren, The Netherlands; 3 Rob Giel Research Center, University Medical Center Groningen, Groningen, The Netherlands; 4 Department of Experimental Psychopathology, University of Groningen, Groningen, The Netherlands; 5 Department of Psychotic Disorders, GGZ Drenthe, Assen, The Netherlands; Catholic University of Sacred Heart of Rome, Italy

## Abstract

Impaired Theory of Mind (ToM) has been repeatedly reported as a feature of psychotic disorders. ToM is crucial in social interactions and for the development of social behavior. It has been suggested that reasoning about the belief of others, requires inhibition of the self-perspective. We investigated the neural correlates of self-inhibition in nineteen low psychosis prone (PP) and eighteen high PP subjects presenting with subclinical features. High PP subjects have a more than tenfold increased risk of developing a schizophrenia-spectrum disorder. Brain activation was measured with functional Magnetic Resonance Imaging during a ToM task differentiating between self-perspective inhibition and belief reasoning. Furthermore, to test underlying inhibitory mechanisms, we included a stop-signal task. We predicted worse behavioral performance for high compared to low PP subjects on both tasks. Moreover, based on previous neuroimaging results, different activation patterns were expected in the inferior frontal gyrus (IFG) in high versus low PP subjects in self-perspective inhibition and simple response inhibition. Results showed increased activation in left IFG during self-perspective inhibition, but not during simple response inhibition, for high PP subjects as compared to low PP subjects. High and low PP subjects showed equal behavioral performance. The results suggest that at a neural level, high PP subjects need more resources for inhibiting the self-perspective, but not for simple motor response inhibition, to equal the performance of low PP subjects. This may reflect a compensatory mechanism, which may no longer be available for patients with schizophrenia-spectrum disorders resulting in ToM impairments.

## Introduction

The capacity to understand other people's mental states or Theory of Mind (ToM) is crucial in social interactions and for the development of social behavior. ToM is a broad concept referring to the ability to understand other people's mental state, the understanding that another person may have a different belief, and the understanding of the others' subsequent behavior [1]. This ToM ability relies upon a wide network of brain areas, most importantly encompassing lateral frontal, medial frontal and temporal areas (see [2] and [3] for a more detailed account of the neurobiological underpinnings of ToM). A large body of research has demonstrated that ToM is often impaired in patients with schizophrenia [4]. Indeed, the overwhelming evidence of ToM impairments in schizophrenia leads to the proposition that impaired ToM may be a trait characteristic of the disorder [4]. Even though patients in the acute phase of the disorder are more impaired than remitted patients, the latter group still demonstrates significant impairments in ToM [5].

ToM problems have not only been observed in people with schizophrenia, but also in subjects with an enhanced risk for schizophrenia, e.g. first-degree relatives of patients [6], [7] and healthy subjects who are prone to psychosis (PP) [8]–[11]. PP, also called schizotypy (though schizotypy refers to a personality trait whereas PP merely implies a vulnerability for psychosis, the terms are used interchangeably), refers to a broad range of sub-clinical experiences and personality characteristics that are related to psychosis in the general population [12], [13]. High PP subjects are thought to have biological and/or cognitive predispositions for the development of psychosis later in life [14], [15]. Consistent with this hypothesis, longitudinal studies have demonstrated that approximately 10% of the high PP subjects will develop a schizophrenia spectrum disorder at a later point [16], [17]. PP may precede an at risk mental state (ARMS) in which clinical symptoms are present, but no transition to psychosis has yet occurred. These individuals show social functioning as well as cognitive deficits, but less severe than patients with a psychotic disorder [18], [19]. Barragan et al. [9] suggested that impairments in ToM may have a developmental nature and are associated with psychotic-like experiences. This would imply that in high PP subjects, such difficulties can be detected in a very early stage. The most important rationale to study ToM in a sub-clinical group without psychosis, was that it allows us to study an important cognitive process known to be involved in psychosis, without confounding factors such as medication, illness duration and institutionalization. Since some studies did not find ToM problems in PP [7] or did find social functioning problems in PP, but no ToM deficits [20], [21], we need other methods to investigate whether such difficulties can be detected in a very early stage. Understanding the underlying neural mechanisms of impairments in ToM at an early stage will provide insights in processes that may induce psychotic decompensation (such as impaired ToM) and provide guidance towards new treatments focussing on social difficulties in schizophrenia.

It has been suggested that observing behavior and emotions in other people automatically activates the representation of such behavior and/or emotions in oneself [22]–[24]. Applying this automatic tendency to assume one's own mental state as a correct model for others when actually unjustified, has been proposed as the underlying cause for impairments in ToM [25]–[27]. Improperly applying this automatic tendency can result in misattributions of mental state and may be induced by a failure to suppress one's own perspective [25]. Such misattributions have been demonstrated in young children [1], [28], adults suffering brain damage [29]–[32] and psychiatric patients (e.g. autism and schizophrenia [33]–[36]). Interestingly, such misattributions have been related to limited inhibitory control [37]. A body of evidence demonstrated that the development of executive functions (like inhibitory control) and ToM [37] is related to the development of the frontal cortex [22]. This may imply that indeed the ability to inhibit the own perspective and general inhibitory control rely upon a similar cortical mechanism.

Building upon the above, Samson et al. [38] proposed a two-component model for ToM comprising (1) a self-perspective inhibition component and (2) a belief reasoning component. Successful self-perspective inhibition was suggested to be a prerequisite for correct reasoning about another person's belief. We recently followed up on Samsons findings and examined the neural basis of this two-component model for ToM [39]. We found that, in line with Samsons results, self-perspective inhibition was mediated by the bilateral inferior frontal gyrus (IFG) with a more prominent role for the left IFG [39], while belief reasoning was mediated by the left superior temporal gyrus (STG), temporoparietal junction (TPJ) and middle temporal gyrus (MTG). Moreover, overlapping areas of activation between self-perspective inhibition and simple response inhibition (using a stop-signal task) were found in the bilateral IFG, but again more prominently in the left IFG, suggesting a common neural inhibitory mechanism for these processes.

In the current study we investigated whether these underlying neural correlates in high PP individuals differ from low PP individuals. Furthermore, we investigated whether this is specific for self-perspective inhibition or whether these differences can also be detected for simple motor inhibitory processes. Since the IFG area appears to be important for self-inhibition and misattributions have been associated with limited inhibitory control, we specifically expected to find differences in activation in the IFG. Furthermore, we expected to find behavioral differences between groups on both self-perspective inhibition as well as on simple motor response inhibition.

By investigating the underlying mechanisms of ToM in PP, we can gain insight into the development of ToM impairments in schizophrenia spectrum disorders. To our knowledge, this is the first study to examine the neural dynamics underlying self-perspective inhibition in PP and, more specifically, its possible relationship with simple inhibitory processes.

## Methods

### Subjects

19 healthy undergraduate students, low PP (9 female, 10 male; mean age 21.6, SD 2.6; same sample as in van der Meer et al. [39]) and 18 healthy undergraduate students, high PP (8 female, 10 male; mean age 19.7, SD 1.9) of the University of Groningen participated in the study. None of the subjects reported a history of psychiatric or neurological disease. All subjects were recruited with the Community Assessment of Psychic Experiences, positive scale (CAPE [40]) and the Beck Depression Inventory (BDI [41]) questionnaires distributed among 600 university students. Subjects were selected from the extremes of the CAPE positive scale, based on willingness to participate, fMRI contra-indications and a score of at least one standard deviation above (high PP group) or below (low PP group) the tested sample mean (mean  = 1.4, SD  = 2). Subjects were only included if their BDI score was not higher than a 10pts cutoff score [41]. Table 1 shows demographic information, BDI and CAPE scores for both groups. All subjects were native Dutch speakers. One of the subjects was left-handed, but since no different brain activation patterns were found, this subject was included in the study. All subjects signed informed consent prior to participation. One subject did not complete the behavioral session. For one of the subjects the CAPE distress score was missing. This study was approved by the local ethics committee (METC) and carried out in accordance with the latest version of the Declaration of Helsinki.

**Table 1:** Demographic information, BDI and CAPE scores for both groups.

	Low PP		High PP	
	mean	SD	mean	SD
**proportion male**	.53	–	.56	–
**Age (years)**	21.42	2.6	20.1	1.87
**CAPE positive** *	1.12	.04	1.8	.15
**CAPE distress** *	3.6	1.6	19.6	.25
**BDI** +	2.42	2.4	6.56	2.67

* **p<0,0001**.

+ controlled for CAPE distress scale.

A chi-square test was used to test for differences in sex between groups, Mann-whitney U tests were performed for age and CAPE scores. Differences in BDI scores were tested with an ANOVA and controlled for the CAPE distress scale.

### Community Assessment of Psychic Experiences (CAPE)

The CAPE questionnaire consists of 42 self-report items measuring lifetime frequency of attenuated psychotic symptoms (on a 4 point scale ranging from 1-never to 4-always). The CAPE has been developed and standardized on a Dutch population, and was previously shown to have good validity and reliability [40]. Questions can be subdivided into three factors: attenuated psychotic symptoms, distress caused by these experiences, and negative symptoms. Since especially positive symptoms of schizophrenia are related to ToM impairments [42], the CAPE positive symptom scale and CAPE distress scale were assessed in this study.

### Procedure

All subjects performed two experimental fMRI tasks, a ToM task and stop-signal task. The order of the tasks was counterbalanced to prevent order effects. The ToM task contained three behavioral control conditions, administered outside the fMRI scanner, and three experimental conditions, administered in the fMRI scanner. Testing for behavioral conditions took place in a quiet environment and lasted approximately 30 minutes for each subject. The fMRI session lasted approximately 60 minutes for each subject.

### Experimental tasks

#### Theory of Mind task

The ToM task consisted of short movie clips (21 sec) and was adapted from Samson et al. [38], [43] and Apperly et al. [29]. The movie clips featured a male and a female actor, a green object and two boxes. The female actor placed a pink note on one of the boxes as a clue for the location of the green object either before or after leaving the room. In her absence the man changed the location of the boxes, resulting in a false belief for the woman upon her return. At the end of the movie clip subjects were required to answer a question either about the belief of the woman or about the location of the object. A detailed description of the task can be found in van der Meer et al. [39].

**Figure 1:**
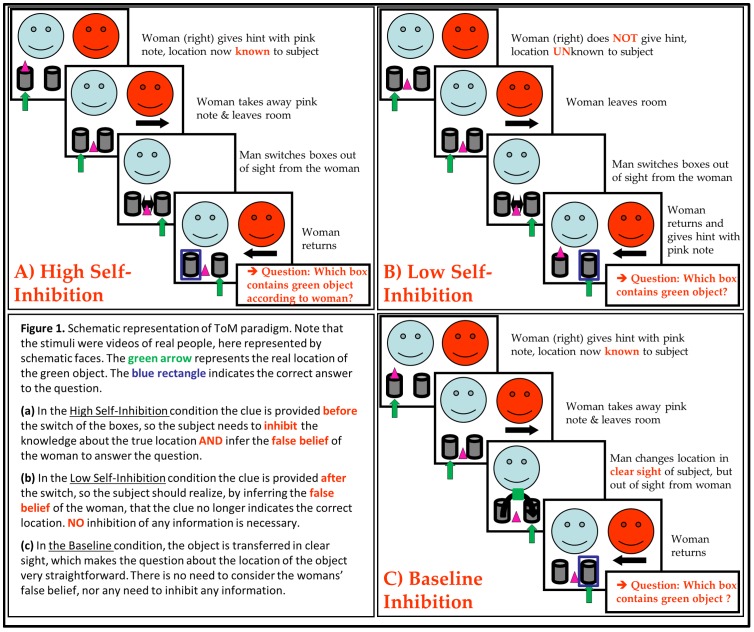
Schematic representation of the Theory of Mind task.

The task contained six conditions. The first three conditions were administered in the MRI scanner (see Figure 1 for a schematic representation); (1) High-Inhibition (HI) requiring inhibition of the subjects' own perspective as well as belief reasoning, (2) Low-Inhibition (LI), requiring only belief reasoning and no self-perspective inhibition, (3) Baseline Control (BC) requiring neither belief reasoning, nor self-perspective inhibition. Though we tried to stay as close as possible to the original movieclips of Samson et al. [38], the movieclips did require slight modification to be able to assess in the MRI scanner (see van der Meer et al. [39] for details about these modifications). For the BC condition we used the c*lue confirmation* videos (see Figure 1) described by Apperly et al. [29].

Besides experimental fMRI conditions, we included three control conditions; (4) Disengage Control, ensuring successful disengagement from one box and subsequent switching of attention to the other box. This is to make sure that incorrect answers on conditions 1–2 are not due to an inability to disengage attention from the incorrect box (DC), (5) Working Memory control, ensuring proper working memory function to make sure incorrect answers on conditions 1–3 are not due to working memory failure (WM) and (6) Strategy Control (SC), controlling for response strategies without full understanding of the task (e.g. always pointing to a different box than the woman). A thorough description of the conditions 4–6 (identical to the conditions *inhibition control, working memory control* and *true belief, respectively*) can be found in Apperly et al. [29].

Conditions 1–3 were administered in the fMRI scanner, conditions 4–6 in a behavioral session. Conditions in the fMRI session entailed 24 movie clips, 12 with the hint on the left box and 12 on the right box. Besides the left/right difference, movieclips within conditions were identical. Six filler items, where questions for HI and LI conditions were switched, were added to make sure the upcoming questions were unpredictable to the subjects. This resulted in a total of 90 trials, administered in five sessions of 18 pseudo randomized trials, to ensure an identical number of trials of each condition per session. In the behavioral session, three blocks of 16 randomized trials were administered, resulting in a total of 48 trials (16 per condition). None of the behavioral conditions required belief reasoning.

#### Stop-signal task

This task was modeled after Rubia et al. [44] and was identical to the task used in our previous study [39]. Subjects were presented with arrows pointing left or right (equally balanced; duration 500 ms) and had to press a key corresponding to the direction of the arrow (go-condition). In the stop-condition, an arrow pointing upward was presented beside the arrow pointing left or right and appeared either just after or simultaneously with the first arrow upon which subjects should hold back their response. The task was programmed so that a maximum of 50% correct would be achieved for the stop trials. See van der Meer et al. [39] for a more detailed description of the task.

### Scanning technique

Subjects were positioned in a 3.0 Tesla whole-body scanner (Philips Intera, Best, NL). The head was kept in position by foam cushions on each side of the head and an elastic band around the head. Stimuli were projected by a beamer onto a screen visible to the subject via a mirror. Responses were given by using the two most outward buttons of a four-button button box to indicate left or right box/arrow.

### Scanning Parameters

Functional images were acquired using a sense-8 head coil. 1309 functional volumes for the ToM paradigm and 280 for the stop-signal paradigm were acquired by T2*-weighted echo planar images consisting of 37 3.5 mm thick axial slices with a 0 mm gap (EPI, TR  = 2.00 s, TE  = 35 ms, flip angle  = 70°, FOV  = 224 mm, 64×64 matrix of 3.5×3.5×3.5 voxels). Slices were acquired interleaved and oriented parallel to the AC–PC plane. A T1-weighted 3D fast-field echo (FFE) anatomical image parallel to the bicommissural plane was acquired covering the whole brain (160 slices; TR  = 25 ms; TE  = 4.6 ms; slice-thickness  = 1 mm; 256×256 matrix; FOV 26 cm; voxel size, 1×1×1 mm).

### fMRI Statistical analyses

FMRI data were converted with MRI-cro (from Philips PAR to Analyze) and analysed using Statistical Parametric Mapping (SPM5), run in MATLAB 7 (The MathWorks Inc., Natick, MA, USA). All functional images were slicetime corrected, realigned, coregistrated with the anatomical T1 image, spatially normalized to standard stereotactic space (MNI T1 template) and spatially smoothed with a 3D isotropic Gaussian kernel (FWHM 10 mm). A high-pass filter of 1.1 times the longest period between two subsequent trials of the same condition was used to filter out systematic low-frequency activation unrelated to the task. For both tasks, only correctly answered items were used in the analyses. Contrasts for the ToM task were made for the question-response period, since the assignment became clear at this moment (during the movieclips, the upcoming question was still unpredictable). To minimize the amount of noise in the signal, a fixed duration of two seconds was used for all ToM trials, since the vast majority of the response times was below two seconds.

For analysis at group level, two-sample T-tests were performed for two contrasts of interest: (1) HI > LI for self-perspective inhibition and (2) ((HI + LI ) > fixation) for belief reasoning. This last contrast was specifically chosen for belief reasoning since our previous work indicated that the BC condition was not a reliable baseline condition due to possible implicit perspective taking processes [39]. A region of interest (ROI) analysis for the IFG was performed for the HI>LI condition, since group differences were expected in this contrast and this region specifically. The ROI was based upon previously published neuroimaging studies on ToM (see File S1 for a detailed description). For the stop-signal task a two sample T-test with the contrast of interest (stop > go) and the same ROI was used to investigate whether differences in activation would be observed in the same areas in simple inhibitory processes as compared to the more complex self-inhibition. Reported activations for (HI > LI) and (stop > go) were FDR corrected (p<.05). All comparisons versus fixation were FWE corrected (p<.05). The minimally activated number of voxels for all contrasts was set to 20.

## Results

### Behavioral data

#### Theory of Mind task

Behavioral data for the self-inhibition task are presented in Table 2. Repeated measures analyses were performed for the behavioral session for *accuracy* and for *reaction times* (RT) with condition (DC, SC & WM) as within subjects factor and group (low vs high PP) as between subjects factor. For *accuracy* no significant effects for condition, group or condition x group interaction were demonstrated (p>.05), which indicates a similar performance for both groups and for all conditions. For RT a significant main effect for condition (F (2, 33)  = 42.7, p<.0001) was found. Pairwise comparisons revealed that RT's for the WM condition were significantly higher than for the DC and the SC condition (p<.0001), but that RT's for DC and SC did not significantly differ (p>.05). No significant main effect for group, nor an interaction effect between group and condition was found. Thus, differences in RT's were observed between conditions, but this effect was similar for both groups.

**Table 2:** Behavioral results ToM and stop-signal task for both groups.

	Healthy Controls			Psychosis Prone		
	Mean RT (sec)	SD (sec)	accuracy (%)	Mean RT (sec)	SD (sec)	accuracy (%)
**ToM task**						
**fmri conditions**						
HI	1.34	.28	97.6	1.34	.20	96.8
LI	.93	.25	97.1	.92	.20	97.5
BC	1.17	.34	99.1	1.22	.20	98.0
**behavioral conditions**						
DC	1.00	.38	99.3	1.00	.30	98.5
SC	.97	.37	97.2	1.03	.26	97.0
WM	1.30	.39	98.3	1.29	.36	97.8
**Stop-signal task**						
go	.49	.10	97	.45	.73	97
stop	–	–	50	–	–	51
go after correct stop	.48	.98	100	.44	.73	97
go after incorrect stop	.50	.10	97	.46	.64	95

Secondly, repeated measures analyses were performed for the fMRI conditions for *accuracy* and for RT's with condition (HI, LI & CC) as within subjects factor and group (low vs high PP) as between subjects factor. This did not reveal any significant effects for condition, group or condition x group interaction with respect to *accuracy* (p>.05). For RT a significant main effect for condition (F (2, 34)  = 99.6, p<.0001) was found, which indicates that RT's differed between conditions. Pairwise comparisons revealed that RT's for HI were significantly higher than for BC and LI (p<.0001), and RT's for BC were significantly higher than for LI (p<.0001). No main effect for group, nor a condition x group interaction was observed for RT's. This indicates that even though a difference in RT's was observed between conditions, this effect was similar for both groups.

### Stop-signal task

Analyses of the behavioral results of the stop-signal task (see Table 2) confirmed the percentage correct mentioned in the method section (mean low PP  = 50%; mean high PP  = 51%), indicating that the algorithm worked properly. A MANOVA with go reaction time (RT) and go accuracy as dependent variables and group (low vs high PP) as an independent variable did not reveal any group differences (p>.05).

### Imaging results

The main effect of the ToM task in low PP only revealed activation in the bilateral IFG for self-inhibition and activation in the STG and TPJ activation for belief reasoning. In addition, simple response inhibition revealed overlapping activation with self-inhibition in the bilateral IFG (see also van der Meer et al. [39]).

### Theory of Mind

**Figure 2:**
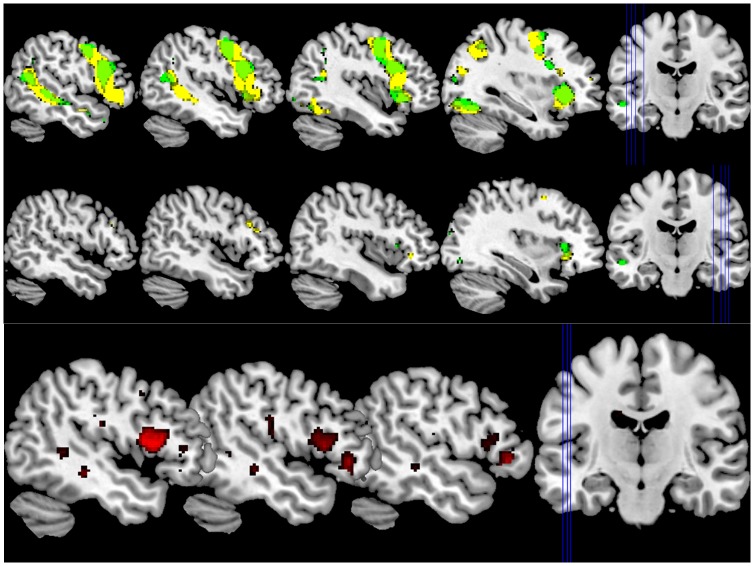
High Inhibition (HI) versus Low Inhibition (LI). Activation patterns for high PP depicted in yellow and for low PP depicted in green. Differential activation pattern between groups depicted in red. TOP: left hemisphere at x-coordinates (MNI-space) −50, −46, −42, −34 (from left to right). MIDDLE: right hemisphere at x-coordinates (MNI-space) 50, 46, 42, 34 (from left to right). BOTTOM: PP > HC, slices represent x-coordinates (MNI-space) −50, −46, −42 (from left to right).

Figure 2 depicts activation patterns for the HI > LI contrast in both groups as well as the difference in activation between groups. Table 3 lists all significant peak activations. As hypothesized, we found significantly more activation in the left IFG for high PP in the HI > LI contrast. An additional whole brain analysis to detect any unexpected differential activation patterns did not reveal any other areas of differential activation in the HI > LI contrast for either low vs high PP subjects or vice versa.

**Table 3:** Peak activations for PP > HC for the ToM contrast HI > LI. For HC> PP no significant peak activations were found.

		MNI coordinates			
Brain region	clustersize (voxels	x	y	z	T-value
*Left IFG*	297	−44	14	10	3.88
		−44	20	14	3.84
		−46	8	12	3.78

The belief reasoning conditions (HI > Fixation and LI > Fixation) revealed left STG, TPJ, MTG, supramarginal gyrus, superior parietal lobe, insula, dMPFC and precuneus activation (see File S2). No group differences were observed for this contrast. Thus, both groups demonstrated similar areas of activation.

#### Stop-signal

The contrast stop > go yielded activation in the bilateral IFG as well as insula, bilateral MTG and post central gyrus. Both groups demonstrated a similar activation pattern (see File S3). No group difference in activation was observed in the region of interest analysis in the IFG. An additional whole brain analysis to detect any unexpected differential activation patterns did not reveal any areas of differential activation between groups.

## Discussion

As hypothesized, the data revealed more activation in the left IFG for high compared to low PP individuals on ToM during self-perspective inhibition, despite equal behavioral performance. This suggests that in order to perform as well as low PP on self-inhibition, recruitment of neural resources is higher in high PP subjects. The low and high PP groups performed equally well on the control ToM conditions, suggesting that differences in the fMRI conditions cannot be due to either problems in working memory, disengagement or response strategies unrelated to the task. No behavioral or functional differences between groups were found in the belief reasoning condition, suggesting that high PP subjects were equally able to reason about others' belief, when no self-perspective inhibition was required. Furthermore, the lack of difference in activation and behavioral performance between groups in the stop signal task suggests that the increased effort in the self-inhibition condition cannot be ascribed to deficits in basic inhibitory processes.

Literature has been equivocal with regard to the performance of high PP on ToM, reporting differences as well as similarities in behavioral performance. A study by Versmissen et al. [7] suggests that the more vulnerable one is for the development of a psychotic disorder, the worse the performance on ToM. Despite the apparent simplicity of our task (the percentage correct for both groups was higher than 97% in all conditions, suggesting a ceiling effect) and equal behavioral performance, high PP subjects showed higher activation of the left IFG during self-inhibition. This finding is especially interesting since it suggests that even though the task was relatively simple for all subjects (all university students), high PP required more resources to reach the same performance in the inhibition of the self-perspective. Interestingly, a study by Kobayashi et al. [45] found increased activation in the left IFG in children relative to adults on a cartoon perspective taking task, despite similar behavioral performance. The authors ascribed the increased activation in the left IFG to a more effortful process. Furthermore, Rapp et al. [46] demonstrated a positive correlation between psychometric schizotypy and activation in the left IFG in an irony comprehesion task, but did not find any behavioral differences between high and low schizotypy. Similarly, Modinos et al. [47] investigated cognitive and emotional ToM using schematic cartoons in high PP individuals. Despite equal behavioral performance, they found increased activation in the left IFG, dorsomedial and anterior prefrontal cortex for high PP in second order ToM but not in first order ToM. Thus, when subjects had to make more complex inferences such as imagining the inference of the other person about yet another person, high PP individuals seemed to need more resources than low PP subjects. When another person's mental state differs from one's own, the self-model may no longer be correct and may require inhibition. Importantly, the design of Modinos et al. [47] did not allow for the separate investigation of self-perspective inhibition. Moreover, the task design of the current study was more similar to real life situations, since they were movieclips of human actors instead of the more abstract cartoons that were used in Modinos et al. [47].

Interestingly, Lee et al. [48] administered a task in schizophrenia patients that beared similarity with the task used in the current study. They distinguished four cartoon conditions including an inhibitory empathy condition, requiring the observer to inhibit the perspective of one of the characters. Thus, this inhibitory empathy condition is comparable to our self-inhibition condition. They found more activation in the right IFG in schizophrenia patients in the inhibitory condition. Even though we found a group difference in left IFG activation, the bilateral IFG was demonstrated to be important for self-perspective inhibition [39]. This seems to suggest that more effort is needed in schizophrenia patients as well as in high PP individuals for the inhibitory component of ToM. In a recent study, Bailey and Henry [49] used exactly the same paradigm as the current study in schizophrenia patients, but did not measure brain activation. Their results showed a trend towards a larger impairment in the HI condition than in the LI condition for schizophrenia patients. Additionally, Jeong et al. [50] found abnormalities in functional as well as anatomical connectivity between the IFG and the superior temporal gyrus, which also has been related to ToM processing [3]. Thus, converging results suggest that one of the core features that may be hampered in schizophrenia as well as in subjects with a predisposition to develop a psychotic disorder, is the inhibition of the own perspective.

An important question is whether such processes are dependent upon more basic inhibitory processes. Even though we found that high PP subjects may need to put in more effort during self-perspective inhibition, such extra inhibitory effort was not found in the simple response inhibition task. This may be due to the lack of contextual cues in our task. Steel et al. [51] demonstrated in a paradigm with richer contextual cues than our paradigm that subjects with positive schizotypy performed worse on a response inhibition task. Such an interpretation is supported by the findings of Barbalat et al. [52], who demonstrated that schizophrenia patients show decreased activation in the bilateral IFG as compared to healthy controls when extra contextual information had to be processed, while no group differences were found for the processing of episodic information. We suggest that in self-perspective inhibition the amount of contextual cues is higher than in simple response inhibition and thus has a higher inhibitory task load. The number of contextual cues in daily life will exceed that of laboratory settings, thus more problems should be expected due to impaired self-perspective inhibition. This reasoning is supported by experiments relating a higher inhibitory task load to decreased performance on ToM tasks in young children [53].

Some limitations with regard to the design of the study should be mentioned. Firstly, both low and high PP groups were selected out of a student population. Thus, all subjects, low and high PP, had a baseline cognitive capacity that may be better than the general population. Selecting high and low PP subjects from the general population may result in more pronounced differences between groups. Furthermore, the design of the current task did not enable us to analyse the activation during the movieclips. Thus, specific processes could not be assessed for the movieclips, but only for the question response period following the movieclips. Finally, the high performance in both groups in terms of accuracy seems to suggest a ceiling effect. Despite this ceiling effect, we found differences in neural activation. This suggest that should we adopt a more complicated paradigm in future research, we may be able to draw firmer conclusions regarding the behavioral and neural performance on ToM of high PP subjects.

In sum, our study suggests that high PP individuals put in more effort when inhibiting the self-perspective. The combined results of self-inhibition and simple motor response inhibition, suggest that this increased effort may lie in the processing of increased contextual cues. Though high PP subjects seem to be able to address a compensatory mechanism for self-inhibition, individuals with more severe psychiatric conditions, such as schizophrenia or autism, may no longer be able to compensate. This may account for their ToM impairments and social disfunctioning.

These results are of relevance, since they suggest that it might not be perspective taking per se in which high PP individuals and schizophrenia patients differ from low PP subject or healthy control subjects, but rather difficulties in the inhibition of one's own perspective. Garety et al. [54] suggested that impaired cognitive and emotional functioning can induce a transition to psychosis for subjects with a vulnerability for psychosis. Thus, investigating the relationship between social functioning, ToM processing, more specifically self-inhibition, and simple inhibitory processing in schizophrenia patients may provide new insight into factors that are predictive of the transition to psychosis in PP individuals.

## Supporting Information

File S1 **ROI analysis.**
(DOC)

File S2 **Belief Reasoning.**
(DOC)

File S3 **Stop Signal.**
(DOC)
