# Supplementary material for: Psychosis-Proneness and Neural Correlates of Self-Inhibition in Theory of Mind
Source: PLoS One. 2013 Jul 18;8(7):e67774. doi: 10.1371/journal.pone.0067774 (PMC3715518; doi:10.1371/journal.pone.0067774)
Supplement: File S1 — ROI analysis. (DOC) [file pone.0067774.s001.doc]

**Supporting information**

**S1 Methods**: ROI analysis


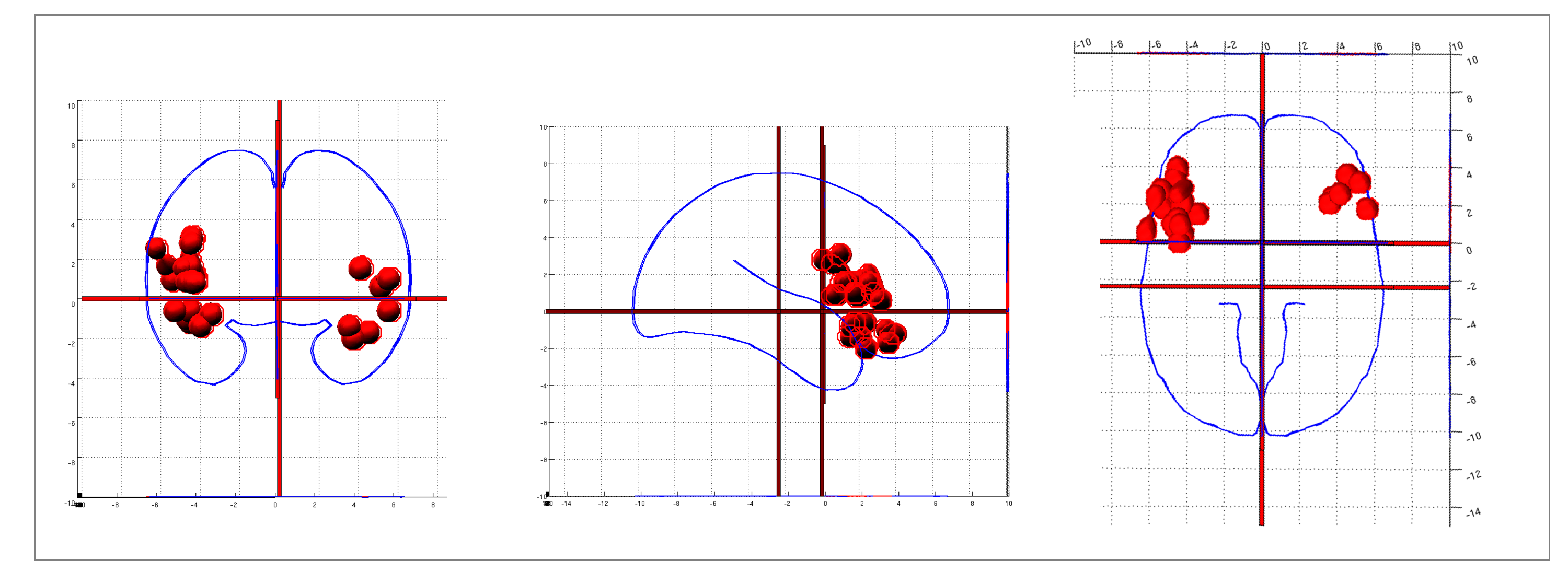
The region of interest was determined by inserting published Talairach peak activations in a ToM contrast in or around the IFG (see table 2) in a 3D template using the Brede toolbox (http://hendrix.imm.dtu.dk/software/brede), implemented in SPM 5. Included peak activations were based on a literature search in pubmed containing the terms [("theory of mind" OR TOM OR "false belief" OR "perspective taking") AND ("inferior frontal" OR IFG)]. Moreover, studies that were included in the meta-analysis on neuroimaging in ToM by Carrington et al.^1^ and that reported activation in or around the IFG were included in the region of interest. Figure 3 depicts the template brain with the peak activations from the literature. Peak activations reported in MNI space were converted to Talairach space in the Brede toolbox. For each included study a separate mask was created by drawing a gaussian kernel of 10 mm around each reported peak voxel. These separate masks were subsequently entered into a data matrix in SPM 5 (using the ImCalc function) to create one single mask, which was then binarized to make it suitable for analysis. This resulted in a mask of 18.5 cm^3^. The number of studies reporting right IFG activation was considerably smaller, which would result in a smaller region of interest in the right hemisphere. To prevent an increase of false positives due to the small size of the region of interest in the right hemisphere, the region of interest that was drawn for the left hemisphere was mirrored for the right hemisphere.

**Figure S1.** Representation of IFG peak activations reported in the ToM literature in coronal, saggital and axial view (from left to right). See table 2 for literature references and peakactivations

**1. Carrington,S.J. & Bailey,A.J. Are there theory of mind regions in the brain? A review of the neuroimaging literature. *Hum. Brain Mapp.* 30, 2313-2335 (2009).**

**Table S1.** Literature reported peak activations for ToM in and around the IFG. Coordinates are in Talairach space, with the exception of the references marked with*. These coordinates were reported in MNI space. These peak activations were transformed into Talairach space in the Brede toolbox.

|  |  | **Coordinates** | | |
| --- | --- | --- | --- | --- |
| **Left/Right** | **Reference** | **x** | **y** | **Z** |
|  |  |  |  |  |
| ***Left*** | *Baron-Cohen et al.^67^* | -46 | 8 | 9 |
|  | *Calarge et al.^68^* | -34 | 15 | -8 |
|  |  | -44 | 0 | 28 |
|  | *David et al.^69^** | -44 | 8 | 34 |
|  | *German et al.^70^* | -39 | 14 | -14 |
|  |  | -48 | 10 | 16 |
|  |  | -45 | 40 | -12 |
|  | *Grezes et al.^71^* | -46 | 34 | -10 |
|  | *Hooker et al.^72^** | -40 | 26 | 12 |
|  |  | -46 | 14 | 8 |
|  | *Hooker et al.^55^** | -50 | 10 | 12 |
|  |  | -38 | 2 | 8 |
|  |  | -54 | 28 | 12 |
|  | *Iacoboni et al.^73^* | -40 | 30 | 6 |
|  |  | -46 | 24 | 24 |
|  | *Mier et al.^74^** | -45 | 24 | -6 |
|  | *Mitchell et al.^75^** | -45 | 12 | 18 |
|  | *Spence et al.^76^* | -52 | 18 | -6 |
|  | *Vogeley et al.^77^* | -62 | 4 | 26 |
|  |  | -56 | 26 | 16 |
|  |  |  |  |  |
| ***Right*** | *Gallagher et al.^78^* | 38 | 24 | -20 |
|  | *German et al^70^* | 56 | 18 | 10 |
|  | *Grezes et al.^71^)* | 52 | 32 | 6 |
|  | *Hooker et al.^72^** | 42 | 26 | 18 |
|  | *Hooker et al.^55^** | 46 | 38 | -18 |
|  | *Spence et al.^76^* | 56 | 18 | -6 |
|  |  |  |  |  |
