# Supplementary material for: Psychosis-Proneness and Neural Correlates of Self-Inhibition in Theory of Mind
Source: PLoS One. 2013 Jul 18;8(7):e67774. doi: 10.1371/journal.pone.0067774 (PMC3715518; doi:10.1371/journal.pone.0067774)
Supplement: File S2 — Belief Reasoning. (DOC) [file pone.0067774.s002.doc]

# Supporting information S2 Belief Reasoning

**
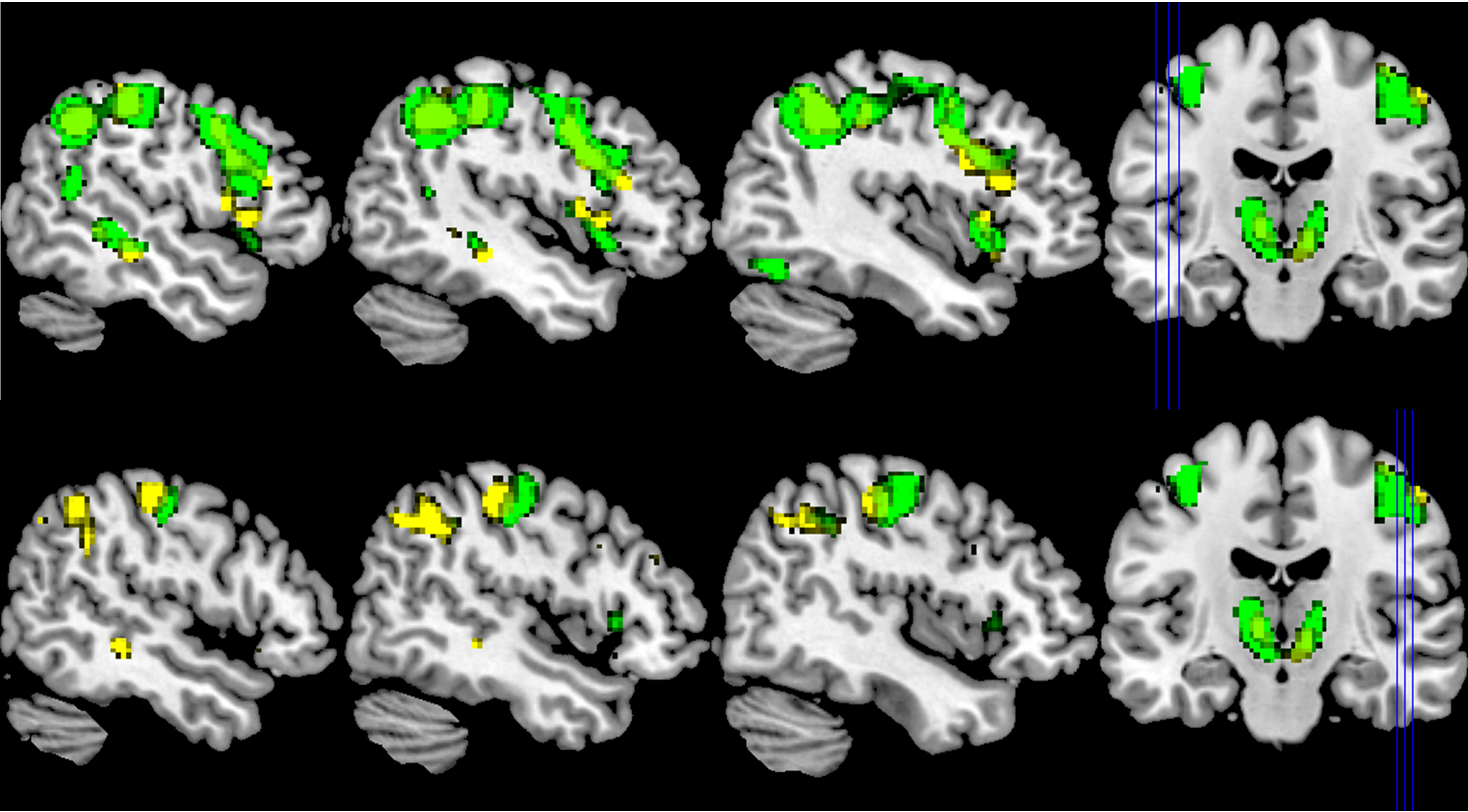
**

**Figure S2a.** High Inhibition (HI) versus fixation. Activation patterns for PP are depicted in yellow, activation patterns for HC are depicted in green. Top: left hemisphere at x-coordinates (MNI-space) -50,-46,-42 (from left to right). Bottom: right hemisphere at x-coordinates (MNI-space) 50, 46, 42 (from left to right).


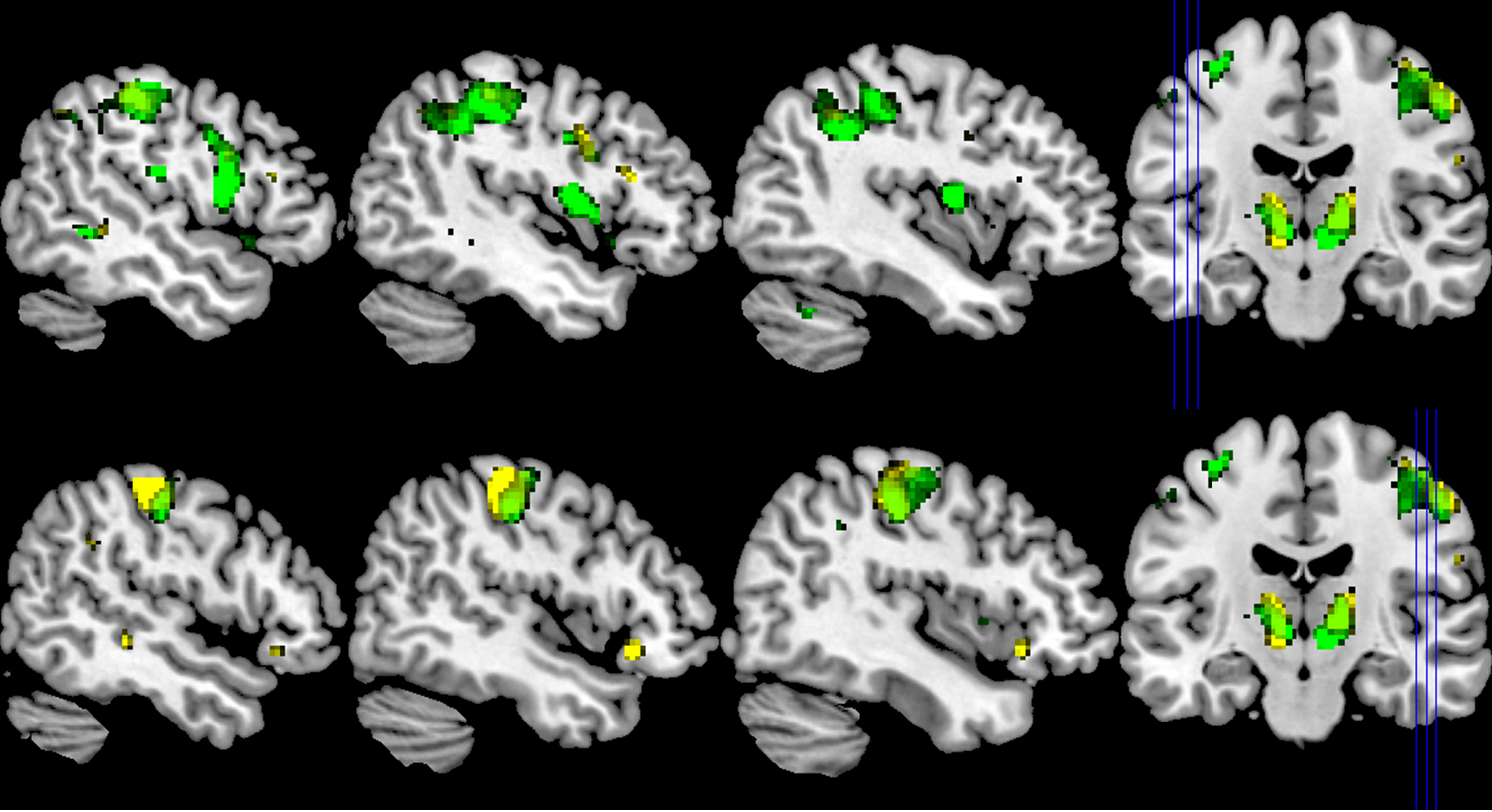


**Figure S2b.** Low Inhibition (LI) versus fixation. Activation patterns for PP are depicted in yellow, activation patterns for HC are depicted in green. Top: left hemisphere at x-coordinates (MNI-space) -50,-46,-42 (from left to right). Bottom: right hemisphere at x-coordinates (MNI-space) 50, 46, 42 (from left to right).

**Table S2a.** HI > fixation and LI > fixation for the HC group

|  |  |  |  | **MNI coordinates** | | |  |
| --- | --- | --- | --- | --- | --- | --- | --- |
| **Condition** | **Brain region** | **left/right** | **clustersize (voxels)** | **x** | **y** | **z** | **T-value** |
|  |  |  |  |  |  |  |  |
| **HI-fixation** | *IFG, insula* | L | 1221 | -30 | 18 | 2 | 15.83 |
|  |  | L |  | -32 | 24 | -10 | 14.78 |
|  |  | L |  | -42 | 10 | 28 | 11.32 |
|  | *inferior, superior parietal cortex* | L | 1046 | -24 | -62 | 42 | 14.13 |
|  |  | L |  | -34 | -54 | 40 | 12.70 |
|  |  | L |  | -24 | -72 | 46 | 11.32 |
|  | *lingual gyrus* | R | 1538 | 4 | -66 | 2 | 13.13 |
|  |  | L |  | -4 | -86 | -6 | 12.19 |
|  |  | R |  | 14 | -72 | 0 | 12.03 |
|  | *superior frontal gyrus* | L | 169 | -36 | 54 | 18 | 12.77 |
|  | *dMPFC* | L | 1404 | -4 | 22 | 54 | 11.94 |
|  |  | L |  | -2 | 30 | 36 | 11.60 |
|  |  | L |  | -4 | 20 | 44 | 11.11 |
|  | *IFG* | R | 170 | 34 | 26 | -6 | 11.70 |
|  | *middle temporal gyrus* | L | 196 | -58 | -42 | -8 | 11.63 |
|  | *inferior parietal lobule* | L | 215 | -50 | -30 | 50 | 11.11 |
|  |  | L |  | -56 | -26 | 46 | 9.26 |
|  | *middle temporal gyrus* | L | 44 | -46 | -30 | -10 | 10.93 |
|  | *midbrain* | L | 333 | -12 | -10 | -2 | 10.36 |
|  |  | R |  | 18 | -8 | -4 | 10.10 |
|  |  | R |  | 6 | -18 | -10 | 10.05 |
|  | *middle temporal gyrus* | R | 112 | 52 | -34 | -8 | 10.23 |
|  |  | R |  | 56 | -42 | -6 | 9.07 |
|  | *superior parietal cortex* | R | 173 | 46 | -24 | 50 | 10.06 |
|  | *inferior parietal lobule, supramarginal gyrus* | R | 270 | 48 | -64 | 42 | 9.79 |
|  |  | R |  | 48 | -50 | 42 | 9.50 |
|  |  | R |  | 50 | -46 | 34 | 8.53 |
|  | *precuneus* | L | 40 | -6 | -68 | 48 | 8.58 |
|  |  |  |  |  |  |  |  |
|  |  |  |  |  |  |  |  |
| **LI-fixation** | *postcentral gyrus* | R | 392 | 46 | -24 | 50 | 12.33 |
|  |  | R |  | 44 | -18 | 60 | 8.66 |
|  | *Insula* | L | 203 | -30 | 26 | -4 | 11.79 |
|  | *Lingual gyrus* | R | 504 | 4 | -84 | -2 | 11.65 |
|  |  | L |  | -4 | -70 | 4 | 10.97 |
|  |  | R |  | 4 | -72 | 4 | 10.48 |
|  | *Middle temporal gyrus* | L | 108 | -58 | -42 | -6 | 10.57 |
|  | *midbrain* | R | 169 | 12 | -10 | 4 | 10.48 |
|  |  | R |  | 16 | -16 | 12 | 8.08 |
|  | *inferior,superior parietal lobe* | L | 311 | -30 | -52 | 40 | 10.05 |
|  |  | L |  | -22 | -64 | 44 | 9.50 |
|  |  | L |  | -42 | -48 | 42 | 7.99 |
|  | *Lingual gyrus* | R | 51 | 22 | -96 | -2 | 9.88 |
|  |  | R |  | 20 | -86 | 0 | 8.43 |
|  | *midbrain* | L | 159 | -16 | -10 | -4 | 9.41 |
|  |  | L |  | -10 | -16 | 6 | 8.68 |
|  |  | L |  | -8 | -18 | -2 | 8.38 |
|  | *postcentral gyrus* | l | 99 | -50 | -30 | 50 | 9.40 |
|  |  | L |  | -48 | -22 | 50 | 7.60 |
|  | *Anterior cingulate gyrus* | L | 67 | -10 | 14 | 34 | 9.26 |
|  |  | L |  | -12 | 24 | 28 | 8.94 |
|  | *Insula* | R | 51 | 44 | 24 | -10 | 8.94 |
|  | *Middle temporal gyrus* | R | 43 | 54 | -32 | -8 | 8.83 |
|  | *putamen* | L | 21 | -30 | 2 | 6 | 8.75 |
|  |  | R | 25 | 30 | -6 | 8 | 8.59 |
|  | *IFG, precentral gyrus* | L | 38 | -48 | 6 | 28 | 8.54 |
|  |  | L |  | -44 | 2 | 36 | 8.53 |
|  | *dMPFC* | L/R | 52 | 0 | 32 | 40 | 8.09 |
|  |  |  |  |  |  |  |  |

**Table S2b** HI > fixation and LI > fixation for the PP group

|  |  |  |  | **MNI coordinates** | | |  |
| --- | --- | --- | --- | --- | --- | --- | --- |
| **Condition** | **Brain region** | **left/right** | **clustersize (voxels)** | **x** | **y** | **z** | **T-value** |
|  |  |  |  |  |  |  |  |
| **HI-fixation** | *IFG, Insula* | L | 1221 | -30 | 18 | 2 | 15.83 |
|  |  | L |  | -32 | 24 | -10 | 14.78 |
|  |  | L |  | -42 | 10 | 28 | 11.32 |
|  | *precuneus, inferior & superior parietal lobe* | L | 1046 | -24 | -62 | 42 | 14.13 |
|  |  | L |  | -34 | -54 | 40 | 12.70 |
|  |  | L |  | -24 | -72 | 46 | 11.32 |
|  | *lingual gyrus* | R | 1538 | 4 | -66 | 2 | 13.13 |
|  |  | L |  | -4 | -86 | -6 | 12.19 |
|  |  | R |  | 14 | -72 | 0 | 12.03 |
|  | *Middle frontal gyrus* | L | 169 | -36 | 54 | 18 | 12.77 |
|  | *dMPFC* | L | 1404 | -4 | 22 | 54 | 11.94 |
|  |  | L |  | -2 | 30 | 36 | 11.60 |
|  |  | L |  | -4 | 20 | 44 | 11.11 |
|  | *IFG* | R | 170 | 34 | 26 | -6 | 11.70 |
|  | *Middle temporal gyrus* | L | 196 | -58 | -42 | -8 | 11.63 |
|  | *inferior parietal lobe* | L | 215 | -50 | -30 | 50 | 11.11 |
|  |  | L |  | -56 | -26 | 46 | 9.26 |
|  | *Middle temporal gyrus* | L | 44 | -46 | -30 | -10 | 10.93 |
|  | *midbrain* | L | 333 | -14 | -10 | -2 | 10.36 |
|  |  | R |  | 18 | -8 | -4 | 10.10 |
|  |  | R |  | 6 | -18 | -10 | 10.05 |
|  | *Middle temporal gyrus* | R | 112 | 52 | -34 | -8 | 10.23 |
|  |  | R |  | 56 | -42 | -6 | 9.07 |
|  | *postcentral gyrus* | R | 173 | 46 | -24 | 50 | 10.06 |
|  | *Angular gyrus, inferior parietal lobe, supramarginal gyrus* | R | 270 | 48 | -64 | 42 | 9.79 |
|  |  | R |  | 48 | -50 | 42 | 9.50 |
|  |  | R |  | 50 | -46 | 34 | 8.53 |
|  | *precuneus* | L | 40 | -6 | -68 | 48 | 8.58 |
|  |  |  |  |  |  |  |  |
|  |  |  |  |  |  |  |  |
| **LI-fixation** | *pre & postcentral gyrus* | R | 392 | 46 | -24 | 50 | 12.33 |
|  |  | R |  | 44 | -18 | 60 | 8.66 |
|  | *Insula* | L | 203 | -30 | 26 | -4 | 11.79 |
|  | *Lingual gyrus* | R | 504 | 4 | -84 | -2 | 11.65 |
|  |  | L |  | -4 | -70 | 4 | 10.97 |
|  |  | R |  | 4 | -72 | 4 | 10.48 |
|  | *middle temporal gyrus* | L | 108 | -58 | -42 | -6 | 10.57 |
|  | *midbrain* | R | 169 | 12 | -10 | 4 | 10.48 |
|  |  | R |  | 16 | -16 | 12 | 8.08 |
|  | *inferior parietal lobe* | L | 311 | -30 | -52 | 40 | 10.05 |
|  |  | L |  | -22 | -64 | 44 | 9.50 |
|  |  | L |  | -42 | -48 | 42 | 7.99 |
|  | *Lingual gyrus* | R | 51 | 22 | -96 | -2 | 9.88 |
|  |  | R |  | 20 | -86 | 0 | 8.43 |
|  | *midbrain* | L | 159 | -16 | -10 | -4 | 9.41 |
|  |  | L |  | -10 | -16 | 6 | 8.68 |
|  |  | L |  | -8 | -18 | -2 | 8.38 |
|  | *postcentral gyrus* | L | 99 | -50 | -30 | 50 | 9.40 |
|  |  | L |  | -48 | -22 | 50 | 7.60 |
|  | *anterior cingulate gyrus* | L | 67 | -10 | 14 | 34 | 9.26 |
|  |  | L |  | -12 | 24 | 28 | 8.94 |
|  | *IFG* | R | 51 | 44 | 24 | -10 | 8.94 |
|  | *middle temporal gyrus* | R | 43 | 54 | -32 | -8 | 8.83 |
|  | *putamen* | L | 21 | -30 | 2 | 6 | 8.75 |
|  | *putamen* | R | 25 | 30 | -6 | 8 | 8.59 |
|  | *IFG* | L | 38 | -48 | 6 | 28 | 8.54 |
|  | *precentral gyrus* | L |  | -44 | 2 | 36 | 8.53 |
|  | *dMPFC* | L/R | 52 | 0 | 32 | 40 | 8.09 |
|  |  |  |  |  |  |  |  |
