# Supplementary material for: Psychosis-Proneness and Neural Correlates of Self-Inhibition in Theory of Mind
Source: PLoS One. 2013 Jul 18;8(7):e67774. doi: 10.1371/journal.pone.0067774 (PMC3715518; doi:10.1371/journal.pone.0067774)
Supplement: File S3 — Stop Signal. (DOC) [file pone.0067774.s003.doc]

# Supporting information S3 Stop Signal


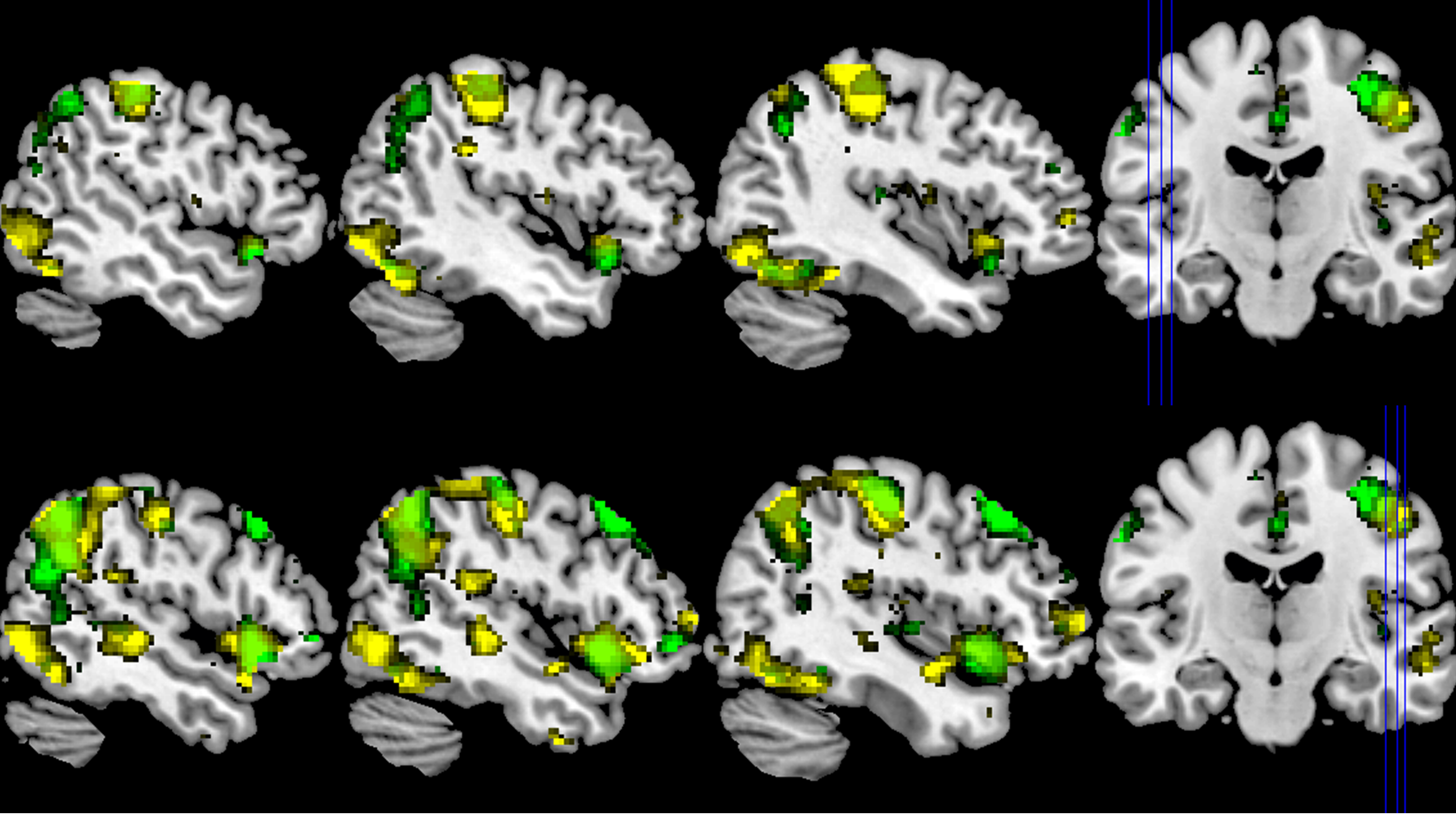


**Figure S3.** Stop versus Go trials. Activation patterns for PP are depicted in yellow, activation patterns for HC are depicted in green. Top: left hemisphere at x-coordinates (MNI-space) -50,-46,-42 (from left to right). Bottom: right hemisphere at x-coordinates (MNI-space) 50, 46, 42 (from left to right).

**Table S3a** Stop > Go HC Group

|  |  |  | **MNI coordinates** | | |  |
| --- | --- | --- | --- | --- | --- | --- |
| **Brain region** | **left/right** | **clustersize (voxels)** | **x** | **y** | **z** | **T-value** |
|  |  |  |  |  |  |  |
| *angular gyrus, postcentral gyrus* | R | 4352 | 56 | -54 | 34 | 10.57 |
|  | R |  | 32 | -42 | 66 | 7.23 |
|  | R |  | 46 | -16 | 48 | 7.14 |
| *post-, precentral gyrus* | L | 857 | -48 | -30 | 54 | 9.05 |
|  | L |  | -36 | -46 | 64 | 5.52 |
|  | L |  | -40 | -22 | 62 | 4.33 |
| *superior, middle frontal gyrus* | R | 616 | 16 | 60 | 26 | 8.15 |
|  | R |  | 38 | 58 | 2 | 6.48 |
|  | R |  | 30 | 46 | 20 | 6.32 |
| *insula, IFG* | R | 1075 | 24 | 24 | -8 | 7.51 |
|  | R |  | 58 | 6 | 0 | 6.56 |
|  | R |  | 50 | 18 | -18 | 6.39 |
| *occipital gyrus, middle temporal gyrus* | L | 1322 | -44 | -76 | -12 | 6.66 |
|  | L |  | -48 | -60 | -18 | 6.22 |
|  | L |  | -60 | -56 | 2 | 6.22 |
| *superior frontal gyrus* | R | 192 | 16 | 34 | 42 | 5.96 |
|  | R |  | 20 | 34 | 50 | 5.67 |
|  | R |  | 14 | 44 | 34 | 4.58 |
| *anterior cingulate gyrus* | L | 218 | -6 | 42 | 12 | 5.70 |
|  | R |  | 4 | 42 | 20 | 4.99 |
|  | R |  | 12 | 34 | 20 | 4.73 |
| *middle temporal gyrus* | L | 101 | -64 | -30 | -6 | 5.40 |
|  | L |  | -66 | -30 | 2 | 4.75 |
|  | L |  | -58 | -22 | -10 | 4.27 |
| *supramarginal gyrus* | L | 200 | -60 | -52 | 38 | 5.38 |
|  | L |  | -64 | -46 | 32 | 4.91 |
|  | L |  | -52 | -64 | 42 | 4.27 |
| *IFG* | L | 161 | -48 | 20 | -8 | 5.28 |
| *inferior parietal lobe* | L | 36 | -46 | -34 | 30 | 5.16 |
| *IFG* | L | 40 | -40 | 52 | 4 | 5.03 |
| *superior temporal gyrus, inferior parietal lobe* | R | 115 | 44 | -34 | 16 | 5.03 |
|  | R |  | 44 | -28 | 22 | 4.68 |
| *caudate* | L | 39 | -16 | 22 | -6 | 4.91 |
| *insula* | R | 87 | 36 | -12 | 14 | 4.80 |
|  | R |  | 34 | -24 | 4 | 4.24 |
| *middle frontal gyrus* | L | 37 | -28 | 40 | 18 | 4.80 |
| *precuneus* | R | 98 | 8 | -52 | 68 | 4.75 |
| *insula* | L | 40 | -26 | 10 | -16 | 4.72 |
| *cerebellum* | L | 91 | -22 | -70 | -34 | 4.69 |
|  | L |  | -26 | -62 | -32 | 4.33 |
| *superior parietal lobe* | L | 56 | -42 | -62 | 52 | 4.51 |
| *cingulate gyrus* | R | 93 | 0 | -2 | 44 | 4.45 |
|  | R |  | 10 | -4 | 42 | 4.14 |
|  | R |  | 4 | -14 | 52 | 4.02 |
| *insula* | L | 40 | -34 | 4 | 12 | 4.39 |
| *cerebellum* | R | 46 | 28 | -42 | -28 | 4.29 |
| *precentral gyrus, insula* | L | 42 | -54 | -2 | 10 | 4.22 |
|  | L |  | -44 | -6 | 12 | 4.21 |
| *cuneus* | L | 26 | -6 | -88 | 10 | 4.05 |
| *lingual gyrus* | R | 32 | 2 | -74 | -4 | 3.86 |

**Table S3b** Stop > Go PP group

|  |  |  | **MNI coordinates** | | |  |
| --- | --- | --- | --- | --- | --- | --- |
| **Brain region** | **left/right** | **clustersize (voxels)** | **x** | **y** | **z** | **T-value** |
|  |  |  |  |  |  |  |
| *inferior parietal lobe, supramarginal gyrus* | R | 1584 | 54 | -48 | 48 | 9.14 |
|  | R |  | 48 | -54 | 48 | 7.37 |
|  | R |  | 56 | -52 | 30 | 7.12 |
| *superior, middlte frontal gyrus* | R | 948 | 18 | 60 | 30 | 7.01 |
|  | R |  | 42 | 28 | 44 | 6.73 |
|  | R |  | 46 | 20 | 48 | 6.37 |
| *cingulate gyrus* | R | 563 | 2 | -32 | 44 | 6.95 |
|  | R |  | 2 | -12 | 42 | 4.98 |
|  | L |  | -2 | 0 | 34 | 3.81 |
| *lingual gyrus, cueus* | R | 146 | 2 | -68 | 8 | 6.89 |
|  | R |  | 4 | -76 | 8 | 4.48 |
|  | L |  | -2 | -74 | -4 | 3.95 |
| *postcentral gyrus, precueus* | R | 1869 | 30 | -22 | 48 | 6.83 |
|  | R |  | 40 | -20 | 50 | 6.72 |
|  | R |  | 6 | -50 | 62 | 6.52 |
| *IFG, insula* | R | 812 | 40 | 22 | -10 | 6.50 |
|  | R |  | 54 | 20 | -4 | 6.19 |
|  | R |  | 58 | 18 | 6 | 5.83 |
| *IFG, insula* | L | 344 | -34 | 14 | -18 | 6.40 |
|  | L |  | -48 | 18 | -10 | 5.53 |
|  | L |  | -36 | -4 | -6 | 4.68 |
| *middle frontal gyrus, IFG* | R | 66 | 48 | 48 | -4 | 5.85 |
|  | R |  | 38 | 48 | 4 | 4.40 |
| *superior frontal gyrus* | L | 77 | -16 | 40 | 48 | 5.75 |
| *postcentral gyrus* | L | 33 | -10 | -30 | 64 | 5.56 |
| *inferior parietal lobe* | L | 433 | -58 | -48 | 46 | 5.23 |
|  | L |  | -50 | -52 | 48 | 5.05 |
|  | L |  | -44 | -60 | 38 | 4.94 |
| *middle occipital gyrus, fusiform gyrus* | L | 206 | -44 | -66 | -16 | 5.15 |
|  | L |  | -40 | -54 | -18 | 4.84 |
|  | L |  | -30 | -66 | -14 | 3.88 |
| *insula* | R | 43 | 40 | -8 | 4 | 4.96 |
|  | R |  | 38 | -20 | 2 | 4.08 |
| *precuneus* | R | 56 | 10 | -68 | 54 | 4.78 |
| *middle frontal gyrus* | L | 50 | -30 | 28 | 42 | 4.68 |
| *middle occipital gyrus, fusiform gyrus* | R | 120 | 36 | -52 | -20 | 4.61 |
|  | R |  | 42 | -64 | -16 | 4.41 |
|  | R |  | 42 | -46 | -14 | 4.28 |
| *precuneus* | R | 83 | 8 | -60 | 38 | 4.58 |
| *cuneus* | R | 65 | 4 | -80 | 32 | 4.55 |
| *supplementory motor area* | L | 57 | -4 | -6 | 56 | 4.40 |
|  | L |  | -8 | -16 | 60 | 4.16 |
| *precuneus* | R | 26 | 8 | -76 | 42 | 3.88 |
